# Automatic Segmentation of Heschl Gyrus and Planum Temporale by MRICloud

**DOI:** 10.1097/ONO.0000000000000056

**Published:** 2024-07-05

**Authors:** Carlos A. Perez-Heydrich, Dominic Padova, Kwame Kutten, Can Ceritoglu, Andreia Faria, J. Tilak Ratnanather, Yuri Agrawal

**Affiliations:** 1 Department of Otolaryngology – Head and Neck Surgery, Johns Hopkins University School of Medicine, Baltimore, MD; 2 Department of Biomedical Engineering, Johns Hopkins University, Center for Imaging Science and Institute for Computational Medicine, Baltimore, MD; 3 Department of Radiology, Johns Hopkins University School of Medicine, Baltimore, MD.

**Keywords:** Hearing Loss, Otology/Neurotology, Radiology

## Abstract

**Objectives:**

This study used a cloud-based program, MRICloud, which parcellates T1 MRI brain scans using a probabilistic classification based on manually labeled multi-atlas, to create a tool to segment Heschl gyrus (HG) and the planum temporale (PT).

**Methods:**

MRICloud is an online platform that can automatically segment structural MRIs into 287 labeled brain regions. A 31-brain multi-atlas was manually resegmented to include tags for the HG and PT. This modified atlas set with additional manually labeled regions of interest acted as a new multi-atlas set and was uploaded to MRICloud. This new method of automated segmentation of HG and PT was then compared to manual segmentation of HG and PT in MRIs of 10 healthy adults using Dice similarity coefficient (DSC), Hausdorff distance (HD), and intraclass correlation coefficient (ICC).

**Results:**

This multi-atlas set was uploaded to MRICloud for public use. When compared to reference manual segmentations of the HG and PT, there was an average DSC for HG and PT of 0.62 ± 0.07, HD of 8.10 ± 3.47 mm, and an ICC for these regions of 0.83 (0.68–0.91), consistent with an appropriate automatic segmentation accuracy.

**Conclusion:**

This multi-atlas can alleviate the manual segmentation effort and the difficulty in choosing an HG and PT anatomical definition. This protocol is limited by the morphology of the MRI scans needed to make the MRICloud atlas set. Future work will apply this multi-atlas to observe MRI changes in hearing-associated disorders.

The primary auditory cortex (PAC) is a major target for afferent auditory information allowing for sound processing and is located within Heschl gyrus (HG) (1–3). The secondary auditory cortex (SAC) is another major target for auditory information, which helps with complex sound processing and is located within the planum temporale (PT) posterior to HG (4,5). Previous studies have shown relationships between volume, gyrification, and other morphological features of HG and PT in hearing-based disorders (1,3,6,7).

The HG is part of the superior temporal gyrus (STG) and crosses the superior temporal plane transversely at the level of the lateral fissure (2,3,8). People frequently have one gyrus on each hemisphere; however, many people can have duplications of HG and have up to 3 gyri per hemisphere (2,3,9). While the PAC is located within this gyrus, its relative location and organization within HG are still under investigation due to this variable gyrification, size, and other morphological characteristics in this region (3,10,11).

The gold standard for localizing regions of interest in MRI scans has remained manual segmentation; however, this can be time-consuming and subject to errors due to the variable gyrification of HG and PT (2,12). To rectify this, some have used probability atlases that would use aggregated data to computationally predict structures based on landmarks without the need for user input (2,8,13).

Programs using probability atlases parcellate the cortical and subcortical regions using a probabilistic classification based on different manually labeled atlases (12,13). While these are valuable, one of the weaknesses of these segmentations is that some of these manually labeled atlases were not specifically tailored for the anatomic variability between people in the STG (13,14). Researchers have previously created tools to localize HG and PT; however, some of these tools must be downloaded and are therefore dependent on the user’s operating system, CPU speed, and software version (12–14).

MRICloud, a cloud-based program, allows the parcellation of uploaded MRI scans regardless of the user’s computing capabilities (15–23). In this study, a multi-atlas of subjects with variable anatomy of HG and PT was segmented to act as the tag to better account for the anatomical variation in patient’s HG and PT. This resegmented multi-atlas was then uploaded to MRICloud and used to segment HG and PT in a sample set of scans. This MRICloud segmentation of the sample set was then compared to a manual segmentation of those regions. This tool could allow for the accurate identification of HG and PT in subjects’ MRI scans without the need for manual segmentation. This tool could also be used in studies such as identifying the differences in HG and PT in individuals with hearing-associated disorders.

## METHODS

### Image Acquisition

Two MRI data sets were used. One was the MRICloud atlas set that consists of 31 scans and the other was a sample data set of 10 scans, which was obtained through the Baltimore Longitudinal Study of Aging (20,23–26).

The MRICloud multi-atlas set was a set of T1-weighted volumetric MRI scans that were acquired in the sagittal plane using a 3T scanner. The sequence used was a T1-weighted image (WI) (magnetization prepared rapid acquisition with gradient echo [MPRAGE]; repetition time [TR] = 2.3–7.3 ms, echo time [TE] = 2.9–3.2 ms, flip angle = 8–9°, image matrix = 240 × 240 to 256 – 256, number of slices =160–240 slices, voxel area = 1.0 × 1.0 mm, slice thickness = 1–1.2 mm).

The sample data set was a set of T1-weighted volumetric MRI scans that were acquired in the sagittal plane using a 3T Philips Achieva scanner at the National Institute on Aging Clinical Research Unit. The sequence used was a T1-WI MPRAGE; TR = 6.5 ms, TE = 3.1 ms, flip angle =8°, image matrix = 256 × 256, number of slices = 170 slices, voxel area = 1.0 × 1.0 mm, and 1.2 mm slice thickness (26).

### Multi-atlas Generation

The original MRICloud 31-brain atlas that contained 287 unique presegmented labels was obtained from MRICloud with subjects ranging from 50 to 90 years old. This multi-atlas was resegmented using ITK-SNAP 3.0 (www.itksnap.org) (27). Specifically, the STG was divided into 4 subregions: the anterior STG, HG, PT, and posterior STG. The criteria for outlining each subregion were based on anatomical landmarks and known functional boundaries. The presegmented image was loaded into ITK-SNAP, and the STG was identified in each slice. The regions of interest were then manually segmented by a single trained researcher to maintain consistency. This resegmented 31-scan multi-atlas contained 293 labels and was reuploaded to MRICloud for public use titled “Adults50-90yrs_293Labels_31atlases_M2_252_V11B.”

### Anatomical Boundaries for HG and PT

This study used previously established guidelines for the boundaries of HG and PT within the STG (Supplemental Table 1, http://links.lww.com/ONO/A27) (2,3,9,11,28,29). For brains with duplications in the HG, there are different types of duplications depending on the depth of Heschl sulcus (HS). This study defined a common stem duplication (CSD) as when the first HS extends for less than 1/2 of the length of HG. This study defined a complete posterior duplication (CPD) as when the first HS extends for greater than 1/2 of the length of HG. For this analysis, both anterior and posterior areas around CSDs have been included in the region designated as HG due to previous research indicating that these regions are more similar to the PAC compared to SAC (2,3,10,12,30,31). In contrast, the posterior part of CPDs was included in the PT in this analysis due to previous studies showing the posterior element of these duplications was more similar to the SAC (2,3,12,30,31). Simplified graphical representation of boundaries used for HG and PT is provided in Supplemental Figure 1, http://links.lww.com/ONO/A28.

### Reference Manual Segmentation

A randomly selected sample set of 10 healthy older adults was collected through the National Institute on Aging Clinical Research Unit. HG and PT were manually segmented in an orthogonal view using ITK-SNAP software. The segmentation was performed by a single trained researcher to maintain consistency.

### MRICloud Segmentation

The sample set of 10 MRIs was then submitted to MRICloud (https://www.mricloud.org/) for segmentation through the newly created pipeline titled “Adults50-90yrs_293Labels_31atlases_M2_252_V11B.”

### Volumetric Analysis

The volume of the reference and automatic segmentations was calculated using the SimpleITK module in Python (32). SimpleITK was used to read the binary segmentation masks and calculate the volume in mm^3^ for each segmentation. The intraclass correlation coefficient (ICC) was then used to compare the agreement of volume of segmentation between the reference and automatic segmentation.


$$\begin{array}{l} ICC=\frac{\sigma^{2}\;between\;reference\;and\;autoamtic\;segmentations\;volumes\;}{\sigma^{2}\;of\;volumes\;within\;segmentation\;groups} \end{array}$$

### Image Overlap Analysis

The overlap between the reference and automatic segmentations in the sample set was quantified using the Dice similarity coefficient (DSC) and the directed Hausdorff distance (HD). The DSC is the proportion of overlap between the 2 segmentation modalities, such that as there is greater overlap between the 2 segmentations, the DSC approaches the value of one. The HD is a measure of the greatest distance between a point in one segmentation to the closest point in the other segmentation. HD describes the distance of nonoverlapping points, such that when all neighboring points on the different segmentations are in close vicinity, the HD value approaches 0 mm. The DSC and HD were calculated for each participant using the SimpleITK toolbox and custom Python scripts (32).

The DSC was calculated as follows:


$$\begin{array}{l} DSC=2\cdot \frac{\left(Reference\;segmentation\cap automatic\;segmentation\right)}{Reference\;segmentation+automatic\;segmentation} \end{array}$$

The HD was calculated using the following equations:


$$\begin{array}{l} HD\left(A,B\right)=\max\left\{h\left(A,B\right),h\left(B,A\right)\right\} \end{array}$$

$$\begin{array}{l} h\left(A,B\right)=\underset{a\in A}{\max}\;\underset{b\in B}{\min}\;a-b \end{array}$$

$$\begin{array}{l} h\left(B,A\right)=\underset{b\in B}{\max}\;\underset{a\in A}{\min}\;b-a \end{array}$$

where *A* = {*a*_1_, *a*_2_, *a*_3_ … *a*_*p*_}, *a* = 3-dimensional coordinates for reference segmentation; *B* = {*b*_1_, *b*_2_, *b*_3_, …*b*_*p*_}, *b* = 3-dimensional coordinates for automatic segmentation.

A volume ratio of the segmentation methods was used to determine the relative sizes of segmentations. It was calculated as the following:


$$\begin{array}{l} Volume\;ratio=\frac{MRICloud\;segmentation\;volume\;in\;mm^{3}}{Manual\;segmentation\;volume\;in\;mm^{3}}. \end{array}$$

### Statistical Analysis

All analyses were performed using STATA version 17 (College Station, TX). The Stata command *icc* was used to perform the ICC test, *regress* was used to perform the linear regression, and *ttest unpaired* was used for the Student *t* tests. A *P* value ≤0.05 was considered statistically significant.

## RESULTS

### Description of Atlas Sets

The MRICloud 31-scans multi-atlas consisted of adults 50–90 years old (mean age:72.5 years, SD: 8.83 years). The multi-atlas had a similar distribution of males and females (15 female, 14 male, and 2 unreported). The sample data set of 10 scans consisted of adults 61–92 years old (mean age: 80.1, SD: 10.3). The sample set was predominantly male (90% male and 10% unreported). Descriptions of the percentages of the number and types of duplications of HG are presented for both groups in Table 1.

**TABLE 1.** Atlas set descriptions

	MRICloud atlas set	Sample data set
Age, range	50–90	61–92
Total scans	31	10
Bilateral single HG	9 (29.0%)	1 (10.0%)
Single-sided duplication	15 (48.4%)	4 (40.0%)
Left-sided duplication	15 (48.4%)	7 (70.0%)
Right-sided duplication	14 (45.2%)	7 (70.0%)
Bilateral duplications	7 (22.6%)	5 (50.0%)
CSD of HG	8 (12.9%^*a*^)	3 (15.0%^*b*^)
CPD of HG	21 (33.9%^*a*^)	11 (55.0%^*b*^)
Total duplications of HG	29 (46.8%^*a*^)	14 (70.0%^*b*^)

^*a*^Percentages out of the total 62 hemispheres of the 31 scans,

^*b*^Percentages out of the 20 hemispheres of the 10 scans.

CPD indicates complete posterior duplication; CSD, common stem duplication; HG, Heschl gyrus.

### Segmentation Comparison

**FIG. 1.**
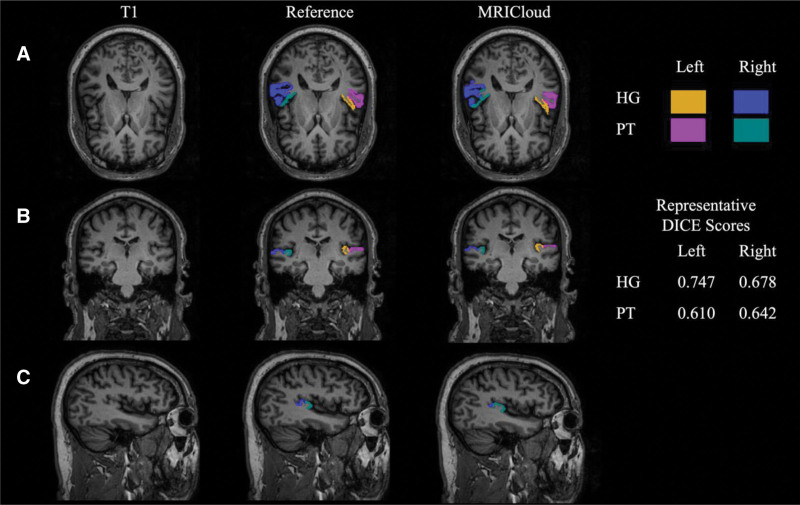
Representative images for manual vs MRICloud segmentation of HG and PT. Subject had a complete posterior duplication (CPD) on the right side and a single HG on the left side. T1 image with both manual and MRICloud segmentation represented in axial, coronal, and sagittal views. Dice scores for overlap are the following: left HG = 0.747, right HG = 0.678, left PT = 0.610, right PT = 0.642. HG indicates Heschl gyrus; PT, planum temporale.

Figure 1 shows an example of the MRICloud segmentation compared to the reference segmentation for HG and PT on both the left and right sides in the validation data set of 10 subjects. The volume of the reference segmentations of the left and right HGs was 1439 ± 476 mm^3^ and 1448 ± 441 mm^3^, respectively (Table 2). The volume of MRICloud segmentations of the left and right HGs was 1327 ± 242 mm^3^ and 1498 ± 385 mm^3^, respectively (Table 2). The absolute ICC for the left HG was 0.79 (0.24–0.95) and 0.95 (0.83–0.99) for the right HG (Table 2). The DSC for the HG comparing the MRICloud segmentation to the reference segmentation was 0.65 ± 0.05 and 0.62 ± 0.06 for the left and right, respectively (Table 2). The HD between the HG segmentations was 6.47 ± 1.94 mm and 7.72 ± 2.46 mm for the left and right, respectively (Table 2).

**TABLE 2.** Manual and automatic segmentation and overlap analysis

	HG		PT		
	Left	Right	Left	Right	Combined
Reference (mm^3^) ± SD	1439 ± 476	1448 ± 441	1776 ± 494	1954 ± 386	6617 ± 1550
MRICloud (mm^3^) ± SD	1327 ± 242	1498 ± 385	1810 ± 298	1966 ± 266	6600 ± 1067
Absolute ICC (CI)	0.794 (0.235–0.948)	0.954 (0.827–0.988)	0.712 (−0.263 to 0.930)	0.200 (−3.40 to 0.814)	0.829 (0.675–0.909)
DSC ± SD	0.645 ± 0.053	0.621 ± 0.060	0.600 ± 0.080	0.600 ± 0.093	0.617 ± 0.071
HD (mm) ± SD	6.47 ± 1.94	7.72 ± 2.46	9.05 ± 3.19	9.17 ± 5.18	8.10 ± 3.47

CI indicates confidence interval; DSC, dice similarity coefficient; HD, Hausdorff distance; HG, Heschl gyrus; ICC, intraclass correlation coefficient; PT, planum temporale.

The volume of the manual segmentations of the left and right PTs was 1776 ± 494 mm^3^ and 1954 ± 386 mm^3^, respectively. The volume of the MRICloud segmentations of the left and right PTs was 1810 ± 298 mm^3^ and 1966 ± 266 mm^3^, respectively. The absolute ICC for the left PT was 0.71 (−0.26 to 0.93) and 0.12 (−3.40 to 0.81) for the right PT. The DSC for the PT comparing the MRICloud segmentation compared to the manual segmentation was 0.60 ± 0.08 and 0.60 ± 0.09 for the left and right PTs, respectively (Table 2). The HD between the PT segmentations was 9.05 ± 3.19 mm and 9.17 ± 5.18 mm for the left and right, respectively (Table 2).

### Segmentation Size Relationship to Segmentations’ Accuracy

**FIG. 2.**
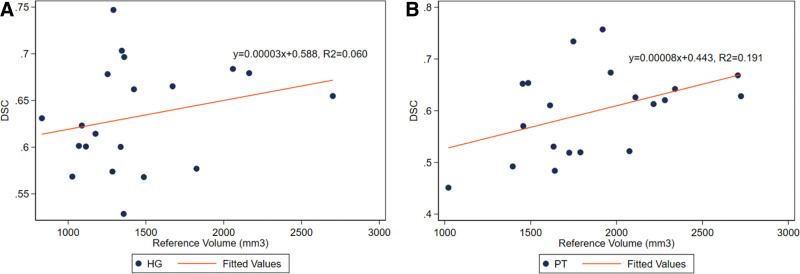
Size dependency of segmentation accuracy. There was no relationship between the DSC of the HG and PT and their reference segmentation volume: *A,* HG (*y* = 0.00003*x* + 0.588, *R*^2^ = 0.060, *P* = 0.298). *B,* PT (*y* = 0.00008*x* + 0.443, *R*^2^ = 0.191, *P* = 0.054). DSC indicates dice similarity coefficient; HG, Heschl gyrus; PT, planum temporale.

**FIG. 3.**
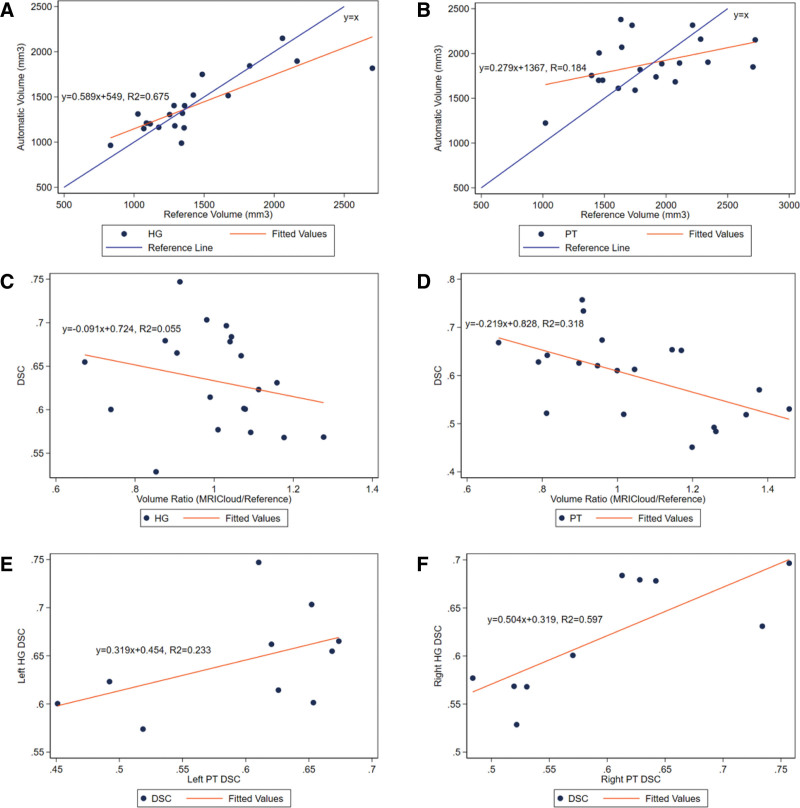
Size estimations of HG and PT affect segmentation quality. There was a strong correlation between the MRICloud segmentation volume and reference segmentation volume for HG but not for the PT: *A,* HG (*y* = 0.598*x* + 549, *R*^2^ = 0.675, *P* < 0.001). *B,* PT (*y* = 0.279*x* + 1367, *R*^2^ = 0.184, *P* = 0.059). The volume ratio of the MRICloud and reference segmentation predicted the segmentation accuracy for the PT but not the HG. *C,* HG (*y* = −0.091*x* + 0.724, *R*^2^ = 0.055, *P* = 0.319). *D,* PT (*y* = −0.219*x* + 0.828, *R*^2^ = 0.318, *P* = 0.010). The DSC was strongly correlated for the right HG and PT but not the left HG and PT. *E,* Left (*y* = 0.319*x* + 0.454, *R*^2^ = 0.233, *P* = 0.158). *F,* Right (*y* = 0.504*x* + 0.319, *R*^2^ = 0.597, *P* = 0.009). DSC indicates dice similarity coefficient; HG, Heschl gyrus; PT, planum temporale.

**FIG. 4.**
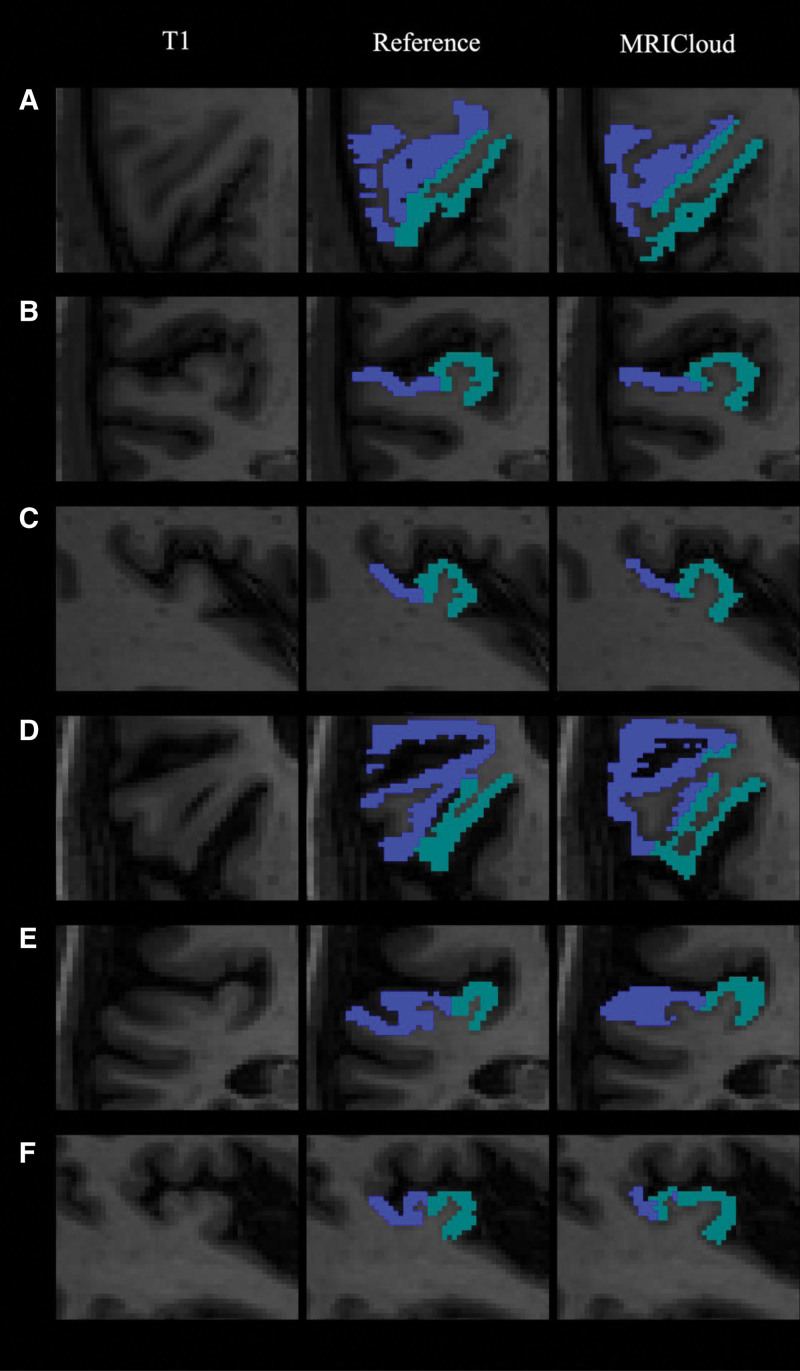
Segmentation images for 2 separate subjects with T1 image and reference segmentation in axial (*A,D*), coronal (*B,E*), and sagittal (*C,F*) views. The dark blue is the right PT and the light blue is right HG. On top is a good segmentation of HG and PT (*A,B,C*) with a DSC of 0.70 for HG and 0.76 for PT. The bottom images are an example of poor segmentation with a DSC of 0.53 for HG and 0.52 for PT. DSC indicates dice similarity coefficient; HG, Heschl gyrus; PT, planum temporale.

Using the DSC as a measure of accuracy or agreement between the reference and automatic segmentation, the DSC of the segmentations of HG and PT did not correlate with the volume of the reference segmentation (HG: *y* = 0.00003*x* + 0.588, *R*^2^ = 0.060, *P* = 0.298; PT: *y* = 0.00008*x* + 0.443, *R*^2^ = 0.191, *P* = 0.054) (Fig. 2A,B). In accordance with the ICC scores reported, the MRICloud segmentations for HG showed a strong association with the volume of the automatic segmentation matching the reference volume (*y* = 0.598*x* + 549, *R*^2^ = 0.675, *P* < 0.0001) (Fig. 3A). However, the volume of the MRICloud segmentation of the PT did not correlate well with the reference volume (*y* = 0.279*x* +1367, *R*^2^ = 0.184, *P* = 0.059) (Fig. 3B). When the MRICloud segmentation underestimated the volume of the PT compared to the reference segmentation, the accuracy of the segmentation was higher; additionally, as the MRICloud segmentation overestimated the PT, the accuracy of the segmentation of the PT was lower (*y* = −0.219*x* + 0.828, *R*^2^ = 0.318, *P* = 0.010) (Fig. 3D). This association of accuracy of the segmentation relative to the estimation of size was not seen in the HG (*y* = −0.091*x* + 0.724, *R*^2^ = 0.055, *P* = 0.319) (Fig. 3C). This overestimation of the PT area affecting the accuracy of the segmentation is seen in the representative images of PT in Fig. 4D,E. When MRICloud was able to segment the right PT appropriately, the right HG was also segmented with greater accuracy (*y* = 0.504*x* + 0.319, *R*^2^ = 0.597, *P* = 0.009) (Fig. 3F). This correlation was also not seen for the left HG and PT (*y* = 0.319*x* + 0.454, *R*^2^ = 0.233, *P* = 0.158) (Fig. 3E).

To demonstrate the effects of duplications of HG and the relative placement of the posterior border placement for HG on the segmentation accuracy of MRICloud, a separate analysis was done incorporating HG and PT into one continuous region called the auditory region. Compared to the individual DSC of HG and PT, the auditory region had a nonsignificant increase in DSC compared to the reference segmentation (0.65 versus 0.62, *P* = 0.077) and a comparable ICC to the reference (0.77 [0.45–0.91] versus 0.83 [0.68–0.91)] (Table 1 and Supplemental Table 2, http://links.lww.com/ONO/A29). Fig. 4A–C shows an appropriate segmentation of the duplication without an overestimation of the PT, while Fig. 4D–F shows MRICloud’s inappropriate incorporation of the complete duplication into HG rather than to the PT in the axial planes on the right side of the brain. Supplemental Figure 2, http://links.lww.com/ONO/A30 shows the same trends but for the left HG and PT.

## DISCUSSION

This study created a novel method to segment the HG and PT, and their associated auditory cortices, using MRICloud, a cloud-based high-throughput neuroinformatic platform for automated brain MRI segmentations.

The overlap of the manual and automated segmentations had moderate agreement for the regions of interest with an average DSC at 0.63 for HG and 0.60 for PT (33). This DSC matches previously automated segmentation methods of HG using probabilistic models that have reported comparable DSCs ranging from 0.3 to 0.7 (13). However, these previously reported segmentations had the advantage that they were recording the entire gyrus and not delineating between the gray and white matter of the gyrus (13). The MRICloud segmentation differentiates the gray and white border, making this technique specifically useful for examining gray matter changes. A study using a similar model of a multi-brain atlas set for automatic segmentation averaged the DSC for cortical regions including PT and obtained a DSC of 0.68 (14). This higher reported DSC may have been seen due to a 45-brain multi-atlas compared to this study’s 31-brain multi-atlas. However, the individual PT DSC was not reported and instead averaged with larger cortical areas, which may lead to a higher DSC compared to this study’s DSC. While there have been automated segmentations that include this region that have reported higher DSC in the range of 0.7–0.9, when considering the small and morphologically distinct HG and PT, an average DSC of 0.617 is appropriate for this region (13,14,33,34).

Certain automated segmentations have detected direct relationships between the size of the region of interest and the accuracy of the segmentation, and that relationship was also observed in our study in reference to the PT (35,36). While the MRICloud and reference segmentations generated volumes that mostly agreed with each other, the ICC showed a discrepancy in the size estimation in the PT, specifically the right PT. The segmentation difference between the MRICloud and reference segmentation may partly be due to some overestimation of the PT region. This was evidenced by when MRICloud oversegmented the PT, there was a trend toward lower accuracy of the segmentation. Furthermore, when the HG and PT regions were incorporated into one continuous region, there was a nonsignificant increase in DSC and a comparable ICC value for that region indicating that the segmentation accuracy of the region may have been affected by the misplacement of the duplications into HG or PT. This reasoning is consistent with the representative images that had lower DSC and poor agreement in volumes showing some misplacement of HG within the defined PT boundaries when there was a duplication. This problem may have been accentuated in this study since this sample data had a higher-than-average HG duplication rate of 70%, while the percentage of duplications in other studies including over 400 subjects ranged between 31% and 64% (3). Additionally, the segmentation differences appear limited due to the small distances between the automated boundaries and the reference boundaries of these structures, as shown by the low HD values for HG and PT bilaterally.

One of the significant limitations of this study is the lack of multiple raters for the manual segmentations. Errors in manual segmentation are common in this highly variably region with some studies reporting high discrepancy between raters (13). In some analyses, 4 raters agreed only about 56% of the time on the ground truth of the total volume of HG in segmentations (13). The quality of segmentations of this multi-atlas set is also limited by the biological diversity present in the atlases used to create the multi-atlas. Specifically, the lack of scans that had more than 2 HG duplications may prove to limit automatic segmentations in subjects with more than 2 HG duplications. The presence of more than 2 duplications of HG is low in the population, but this lack of representation in the multi-atlas may still limit the ability of the multi-atlas to properly segment this anatomical variation (3). Regarding appropriate representation, as the multi-atlas was verified, the randomly selected sample set did mostly match the MRICloud atlas set in terms of the presence of both CSD and CPD. The duplication rate of the HG at 46.8% in the atlas set is within the normal range seen in the general population but lower than the sample set at 70% (3). Another limitation of the study regarding duplications is the exclusion of CPDs from the HG tag, due to disagreement between studies about whether complete duplications are more similar to the PAC rather than the SAC (2,3,10,30,31). Therefore, studies that intend to use this tool for analysis of HG and the marker of the PAC should be aware that the HG tag in this study may not encapsulate all the PAC (2,3,10,30,31).

One of the strengths of this project is the fact that we achieved a reasonably accurate segmentation of this highly variable region. Also, this tool was added to a cloud-based service that is now able to create a contiguous segmentation of the brain with 293 labels and provide information such as volumes for the regions of interest. This tool can now segment many T1 MRIs regardless of the user’s computer capabilities with a tag for both HG and PT.

## CONCLUSION

This study created a segmentation protocol of the HG and PT and their associated primary and secondary auditory cortices, which is based on a probabilistic classification hosted on MRICloud, a web-based service. The multi-atlas set created is effective at segmenting areas associated with hearing within the STG. This tool can act as a preprocessing of MRIs for use but may still require visual inspection to confirm the region of interest boundaries. Future work will apply this multi-atlas to the study of changes in these regions in hearing-associated diseases.

## FUNDING SOURCES

This work used Stampede2 cluster at TACC and Expanse cluster at SDSC through allocation “ASC140026” (Computational Anatomy Gateway) from the Advanced Cyberinfrastructure Coordination Ecosystem: Services & Support (ACCESS) program, which is supported by National Science Foundation grants #2138259, #2138286, #2138307, #2137603, and #2138296. This work was also supported by National Institute of Heath grant #P41-EB03177.

## CONFLICT OF INTEREST STATEMENT

None declared.

## DATA AVAILABILITY STATEMENT

The datasets generated during and/or analyzed during the current study are not publicly available but are available from the corresponding author on reasonable request.

## Supplementary Material


