# Supplementary material for: Automatic Segmentation of Heschl Gyrus and Planum Temporale by MRICloud
Source: Otol Neurotol Open. 2024 Jul 5;4(3):e056. doi: 10.1097/ONO.0000000000000056 (PMC11424062; doi:10.1097/ONO.0000000000000056)
Supplement: Supplementary file 1 [file on9-4-e056-s001.pdf]

**Supplemental Table 1.** Description of anatomical boundaries for HG and PT.

| | Heschl's Gyrus | Planum Temporale |
|---|---|---|
| Anterior | The first transverse sulcus (TS) that unites medially with the circular sulcus of the insula | I. Single HG: Heschl's Sulcus<br>II. CPD: The posterior limit of the most anterior HG |
| Posterior | I. Single HG: Heschl's Sulcus (HS)<br>II. CPD: The posterior limit of the most anterior HG | The vertical ascending terminal segment of the Sylvian fissure |
| Medial | Line from medial end of TS to medial end of HS | Circular Sulcus |
| Lateral | Extending lines from TS and HS to the lateral border of the temporal plane | Superolateral margin of the superior temporal gyrus |
| Superior | Sylvian Fissure | Inferior perisylvian gray matter |
| Inferior | Line from depth of HS to the notch created by meeting of the superior surface of the HG and its stem | The white matter of the inferior perisylvian gray matter |
