# Supplementary material for: Automatic Segmentation of Heschl Gyrus and Planum Temporale by MRICloud
Source: Otol Neurotol Open. 2024 Jul 5;4(3):e056. doi: 10.1097/ONO.0000000000000056 (PMC11424062; doi:10.1097/ONO.0000000000000056)

Supplemental Figure 1. Simplified graphical representation of single HG compared to CSD and CPD in an axial MRI view. The HG is in light blue and the PT is in dark blue. The single HG is bounded posteriorly by the HS and then the PT (A). In a CSD, the first HG is bounded posteriorly by the first HS that extends less than half of the total length of HG. In this case the duplication included in HG and the second HS divides the CSD from the PT. In a CPD, the first HG is bounded posteriorly by the first HS that extends greater than half of the total length of HG and therefore the posterior duplication is included in the PT (C).

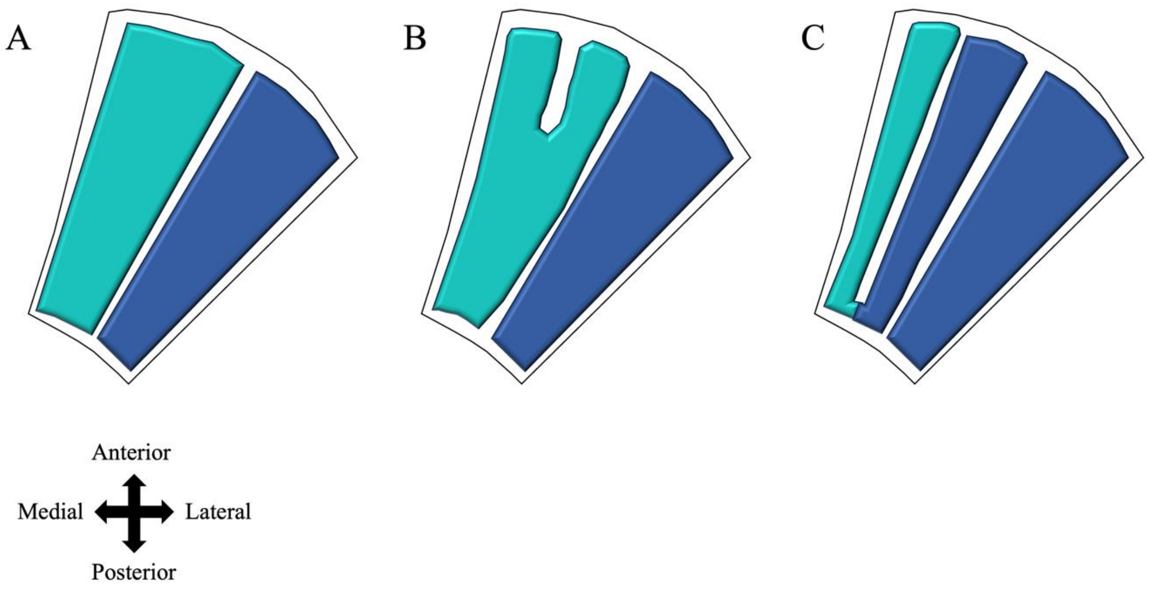

Supplement: Supplementary file 2 [file on9-4-e056-s002.pdf]
