# Supplementary material for: Automatic Segmentation of Heschl Gyrus and Planum Temporale by MRICloud
Source: Otol Neurotol Open. 2024 Jul 5;4(3):e056. doi: 10.1097/ONO.0000000000000056 (PMC11424062; doi:10.1097/ONO.0000000000000056)
Supplement: Supplementary file 3 [file on9-4-e056-s003.pdf]

Supplemental Table 2. Combined Auditory Region Assessment.

	Auditory Region L	Auditory Region R	Combined
Reference Volume (mm^3) ± SD	3214 ± 897	3400 ± 733	6617 ± 1550
MRICloud Volume (mm^3) ± SD	3135 ± 481	3463 ± 628	6600 ± 1067
ICC [CI]	0.716 [-0.651-0.945]	0.801 [0.162-0.951]	0.773 [0.416-0.911]
Individual Regions DSC	0.621 ± 0.057	0.611 ± 0.072	0.617 ± 0.071
Auditory Region DSC	0.651 ± 0.048	0.648 ± 0.072	0.649 ± 0.060
Individual vs Combined Region DSC significance (p-value)	0.214	0.261	.077

SD: Standard Deviation, CI: Confidence Interval
