# Supplementary material for: Automatic Segmentation of Heschl Gyrus and Planum Temporale by MRICloud
Source: Otol Neurotol Open. 2024 Jul 5;4(3):e056. doi: 10.1097/ONO.0000000000000056 (PMC11424062; doi:10.1097/ONO.0000000000000056)
Supplement: Supplementary file 4 [file on9-4-e056-s004.pdf]

Supplemental Figure 2 Segmentation images for two separate subjects with T1 image and reference segmentation in axial (A,D), coronal (B,E), and sagittal (C,F) view. The dark pink is the left PT and the gold is left HG. On top is a good segmentation of HG and PT(A,B,C) with a DSC of 0.67 for HG and 0.67 for PT. The bottom images are an example of a poor segmentation with a DSC of 0.62 for HG and 0.49 for PT.

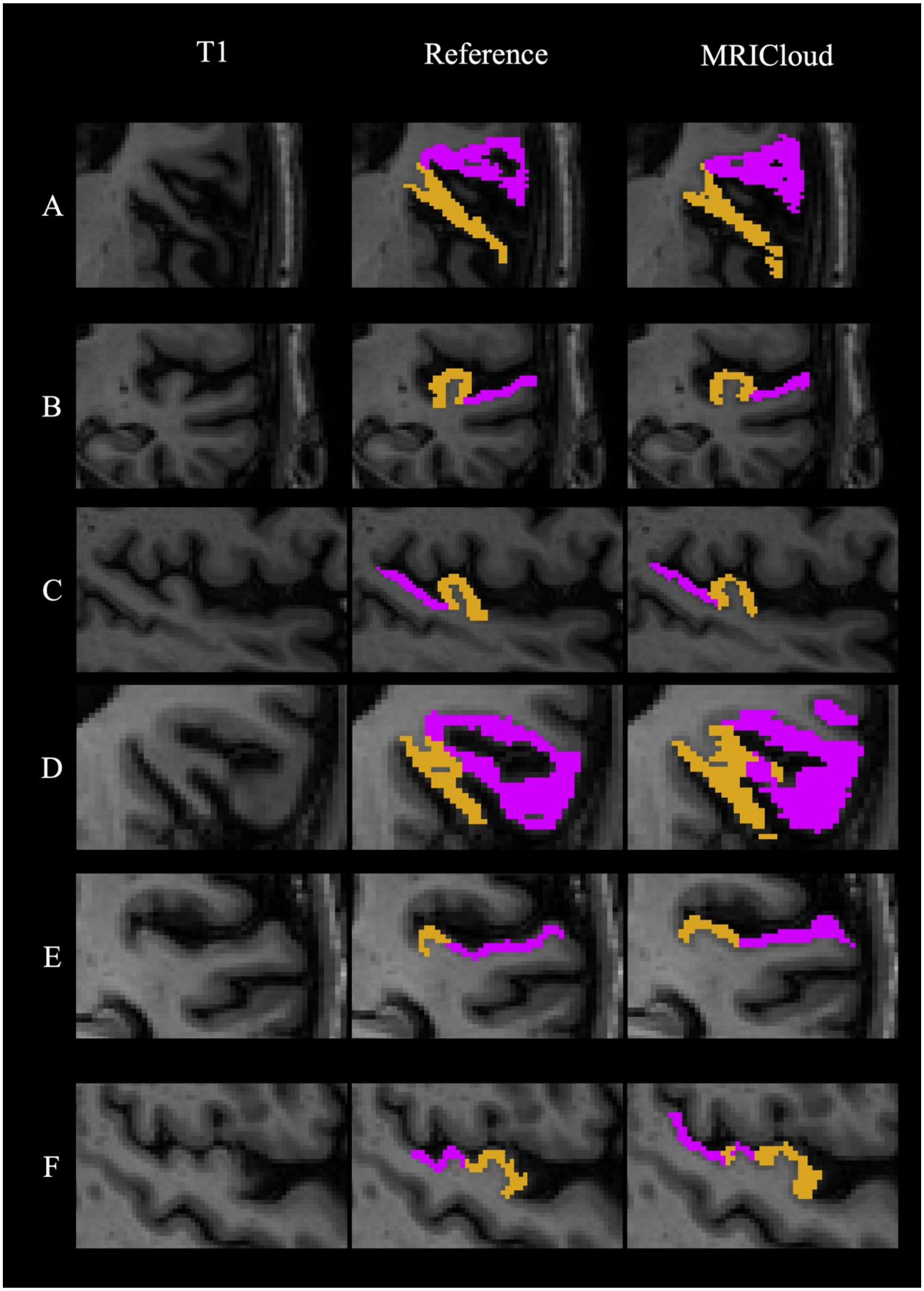
T1
Reference
MRICloud
A
B
C
D
E
F
